# Cyclic Behavior and Stress–Strain Model of Nano-SiO_2_-Modified Recycled Aggregate Concrete

**DOI:** 10.3390/ma17051180

**Published:** 2024-03-03

**Authors:** Yingwu Zhou, Wenzhuo Xu, Wenwei Lin, Jiahao Zhuang, Feng Xing, Rui Hu

**Affiliations:** 1 Guangdong Provincial Key Laboratory of Durability for Marine Civil Engineering, Shenzhen University, Shenzhen 518060, China; ywzhou@szu.edu.cn (Y.Z.); 2100471029@email.szu.edu.cn (W.X.); 2150471009@email.szu.edu.cn (W.L.); 2110474047@email.szu.edu.cn (J.Z.); xingf@szu.edu.cn (F.X.); 2 School of Mechanics and Construction Engineering, Jinan University, Guangzhou 510632, China

**Keywords:** recycled aggregate concrete, nano-SiO_2_, cyclic loading, skeleton curve, stress–strain model

## Abstract

Recycled aggregate concrete (RAC) possesses different mechanical properties than ordinary concrete because of inherent faults in recycled aggregates (RAs), such as the old interfacial transition zone (ITZ). However, the application of nano-SiO_2_ presents an effective methodology to enhance the quality of RA. In this study, nano-SiO_2_-modified recycled aggregate (SRA) was used to replace natural aggregate (NA), and the stress–strain relationships and cyclic behavior of nano-SiO_2_-modified recycled aggregate concrete (SRAC) with different SRA replacement rates were investigated. After evaluating the skeleton curve of SRAC specimens, the existing constitutive models were compared. Additionally, the study also proposed a stress–strain model designed to predict the mechanical behavior of concrete in relation to the SRA replacement rate. The results show that compared with RAC, the axial compressive strength of SRAC specimens showed increases of 40.27%, 29.21%, 26.55%, 16.37%, and 8.41% at specific SRA replacement rates of 0%, 30%, 50%, 70%, and 100%, respectively. Moreover, the study found that the Guo model’s calculated results can accurately predict the skeleton curves of SRAC specimens.

## 1. Introduction

As global industrialization and urbanization have accelerated, the amount of waste generated during building and demolition has increased correspondingly, causing serious environmental problems. The construction industry suffers the burden of more than 25% of global carbon dioxide emissions, which emphasizes the industry’s significant impact on the environment [1,2,3]. In the world, concrete contributes approximately thirty to forty percent of all construction-related waste [4]. As a result, over the last few decades, intense research has been directed towards improving the sustainability index of the construction industry. NA is used extensively during the concrete-making process, placing a great deal of strain on the environment’s resources. Therefore, replacing NA with RA has become a research subject in the field of building materials [5,6].

Recycle aggregate is a green building material obtained from the crushing of waste concrete in construction and demolition. Previous research reveals that RAs have inferior mechanical performance compared to NA [7]. The difference is mostly caused by elements like higher porosity, increased water absorption, surface crack propagation, and the presence of residual mortar. These characteristics negatively impact the structural integrity and durability of both fresh and hardened concrete mixes containing RA. Additionally, the old interfacial transition zone (ITZ), which consists of the original natural aggregates and old mortar, has a significant negative impact on RA performance [8]. In order to enhance the quality of RA, researchers are currently trying innovative methods of treatment [9,10,11]. Wu et al. [10] demonstrated that old mortar can be effectively separated from RA surfaces by thermal treatment at 700 °C. Wang et al. [12] tested modifying RA using different concentrations of acetic acid and soaking times with acetic acid. This soaking procedure effectively removed the cement mortar from RA surfaces, eliminating flaws and enhancing the mechanics of RAC. The optimal conditions for acetic acid soaking were found to be a concentration of no more than 3% and a soaking period of one day. However, the acid immersion method can cause new cracks to appear on the aggregate and weaken the durability of the concrete [13]. So, it is necessary to develop methods that enhance the surface mortar and improve the performance of the interfacial transition zone (ITZ) without removing the old mortar. Jalilifar et al. [14] discuss a method of strengthening the old mortar in recycled aggregates by soaking them in a volcanic ash slurry. The study found that using 10% silica fume in recycled concrete significantly reduced porosity and compacted the ITZ through the bridging of hydration products. The ITZ of this treated concrete was found to be very similar to that of conventional concrete. Krzywinski et al. [15] prepared green epoxy resin coatings using recycled fine aggregate. The study found that epoxy resin coatings modified with 20% natural fine aggregate and 80% recycled fine aggregate showed the greatest improvement in mechanical properties.

**Figure 1:**
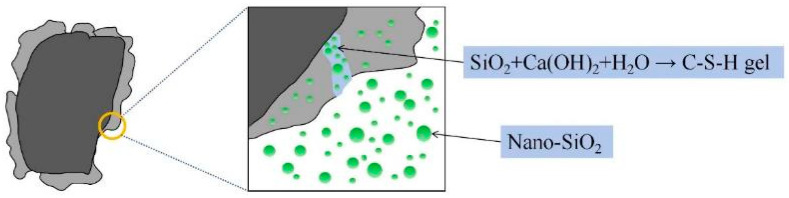
The mechanism of nano-SiO_2_-modified recycled aggregate.

In recent years, researchers have attempted to use nano-SiO_2_ to treat RA. The basic principle of using nano-SiO_2_ for the modification and enhancement of recycled aggregate is shown in Figure 1.

According to studies [16,17], the surface area and pozzolanic activity of nano-SiO_2_ can expedite the reaction with the cement hydration product CH, forming C-S-H gel. In the surface of the RA, this leads to the formation of a denser ITZ. Therefore, the previous research indicated that the incorporation of nano-SiO_2_ in RAC can effectively enhance its mechanical properties, durability, and so on [18]. Alhawat et al. [19] studied the effect of nano-SiO_2_ incorporation on the bonding performance of RAC and found that incorporating nano-SiO_2_ with a cement replacement mass fraction of 1.5% raised the bond strength of RCA from 8% to 21%. Furthermore, because of its extremely small particle size, nano-SiO_2_ can efficiently fill pores and microcracks throughout the immersion process by entering the interior of RA [20]. Zhao et al. [21] pointed out through experiments that 2% nano-SiO_2_ solution and a 48 h soaking period can achieve the most efficient modification in RA. Under these optimal conditions, compared to RAC, the 28-day compressive strength, flexural strength, and elastic modulus of nano-SiO_2_ increased by 31.8%, 33.2%, and 89.6%, respectively. Therefore, in a nano-SiO2 solution is a more preferable modification method. In addition to being easy to use and reasonably priced, this modification method may be applied to large-scale processing. This modification method helps to transform waste aggregates into usable construction materials, thereby reducing the demand for virgin materials and effectively addressing the issue of waste utilization.

Extending the work of earlier studies, this paper employs a method of soaking RA in a 2 wt% nano-SiO_2_ solution to modify it. The purpose of this research is to analyze the cyclic behavior of SRAC at various SRA replacement rates, while also assessing the applicability of existing constitutive models to SRAC. Additionally, a stress–strain model for SRAC with various SRA replacement rates has also been established.

## 2. Materials and Methods

**Figure 2:**
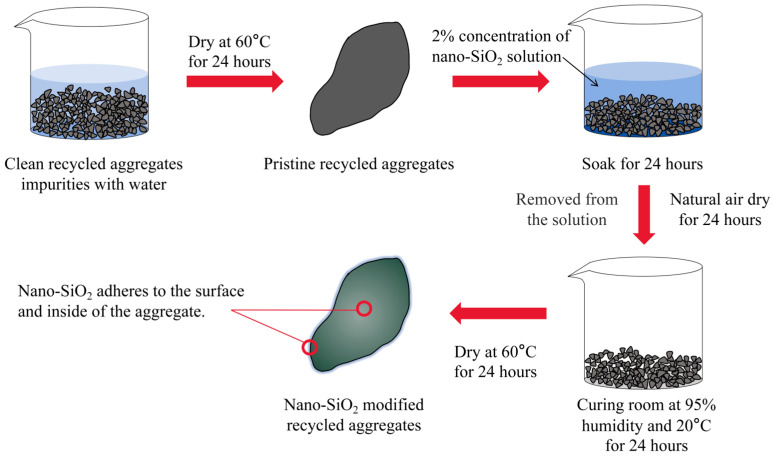
The process of nano-SiO_2_ modification.

P.O 42.5 cement was selected for this experiment, and its basic properties are shown in Table 1. The sand was natural river sand, and the aggregate included both NA and RA. RA was obtained by crushing support beams from a construction pit project in Shenzhen, China. SRA was obtained by modifying RA with nano-SiO_2_ solution. The nano-SiO_2_ solution was sourced from Zhejiang Yuda Chemical Co., Ltd. in Shaoxing, China, and Table 2 provides detailed information about the nano-SiO_2_ solution. The modification of the recycled aggregates with nano-SiO_2_ is illustrated in Figure 2: First, the impurities are cleaned from RA using water, and then dried at 60 °C for 24 h. RA is soaked for 24 h in a 2% nano-SiO_2_ solution. After being soaked and removed from the solution, RA is air-dried in a lab for 24 h, then placed in a standard curing room at 95% humidity and 20 °C for another 24 h. Finally, the cured RA is placed in an oven set to 60 °C for 24 h to obtain SRA.

Table 3 displays the measured performance of aggregates. The water absorption rate is measured according to the method outlined in ASTM C642-13 [22], while the crushing value is measured according to the method specified in ASTM C136 [23]. It is evident that RA has a much higher water absorption and crushing index than NA, which is explained by the old mortar that is still adhering to RA. After nano-SiO_2_ treatment, the performance of RA has been improved, but there is still a significant gap compared to NA.

**Figure 3:**
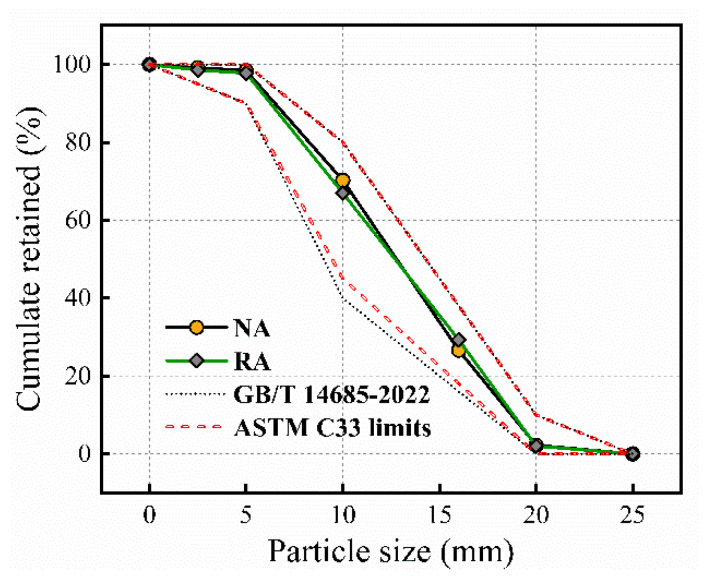
The gradation of aggregates.

The aggregate gradation data in Figure 3 were tested according to the measuring method from ASTM C131-14 [24]. The graph demonstrates a clear alignment of all these aggregates with established code standards [25,26]. The consistency in gradation across these materials is evident from the graphical representation, reaffirming their compliance with industry specifications. This comprehensive illustration aids in confirming that the SRA, RA, and NA utilized in the experiment adhere to the prescribed grading standards, ensuring a reliable and standardized foundation for the research.

Table 4 displays the mix proportions for test specimens. The compensation water is calculated according to the water absorption and dosage of SRA. Four different SRA replacement rates (30%, 50%, 70%, and 100%) of the SRAC specimens were considered for six groups of test specimens, while the other two specimens (N-100 represents natural aggregate concrete, and R-100 represents recycled aggregate concrete) serves as the reference concrete. Each group consists of three cylinder specimens measuring 150 mm in radius and 300 mm in height. Every specimen mixing procedure occurred in the laboratory environment. Test specimens were treated for 28 days.

**Figure 4:**
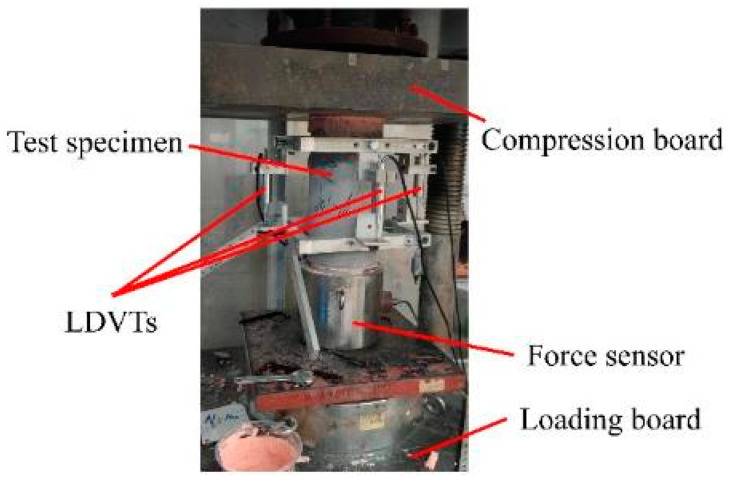
Test setup.

All specimens were tested using the MTS 300 T microcomputer-controlled servo press, depicted in Figure 4. Linear variable displacement transducers (LVDTs) measured the longitudinal displacements, while the digital image correlation (DIC) system was used to measure the strain on the surface of the concrete to ensure the accuracy of the longitudinal displacement of the concrete. The force sensor determined the axial compressive force applied to the specimen. The elastic modulus of each specimen was calculated according to GB/T 50081-2019 [27]. According to Hooke’s law, the elastic modulus can be determined by calculating the ratio of stress to strain before the stress reaches one-third of the peak stress. The peak strain of a specimen is obtained by recording the strain experienced by the specimen when it reaches peak stress during the testing process.

**Figure 5:**
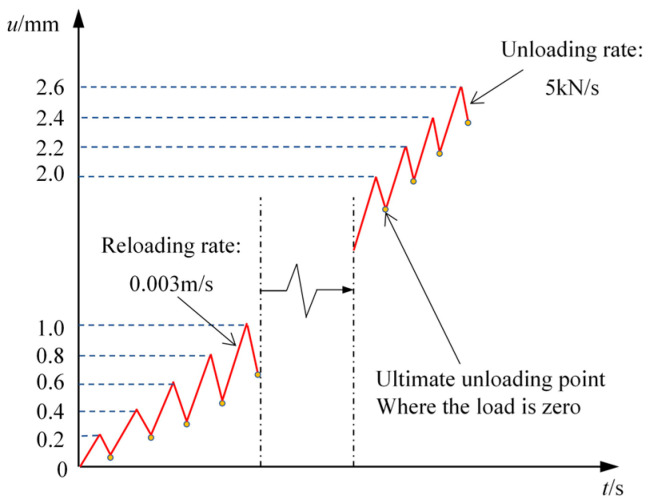
Loading regime diagram.

The displacement loading rate for the monotonic test was maintained at 0.02 mm/s, which translates to a strain rate of 67 × 10^−6^/s. In the cyclic test, the maximum displacement rises by around 0.2 mm with each cycle, and the load is applied repeatedly until the specimens fail. The specimen was first loaded at a strain rate of 67 × 10^−6^/s for each cycle, and it was subsequently unloaded at a rate of 5 kN/s till 0. Figure 5 exhibits the loading regime for the cyclic compression test.

## 3. Results

### 3.1. Failure Modes

In the early stages of stress, there were no obvious cracks on the specimen’s surface. The specimen surface initially produced longitudinal microcracks, which gradually turned into inclined cracks as the load increased. The specimen eventually collapsed due to the inclined cracks becoming wider and longer after they reached their maximal stress. During loading and unloading cycles, it is noteworthy to observe that the cracks first developed and subsequently slowly closed. The cracks did not entirely close because of the aggregates’ interlocking effect.

**Figure 6:**
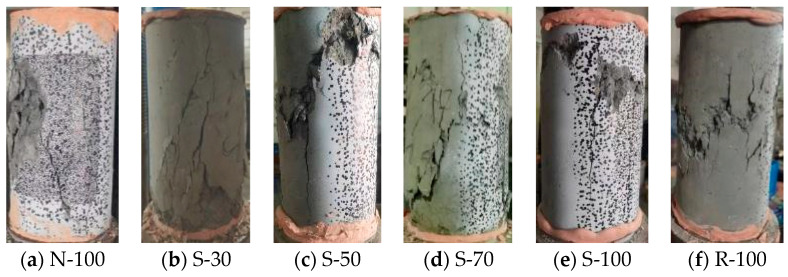
Failure modes of test specimens. (**a**) N-100; (**b**) S-30; (**c**) S-50; (**d**) S-70; (**e**) S-100; (**f**) R-100.

Figure 6 illustrates the failure mechanisms shown by each specimen under cyclic stress. All specimens were finally crushed, resulting in longitudinal cracks on the surface. The specimen N-100 exhibited a major inclined crack on its surface. Conversely, microscopic angled cracks occurred significantly along the surface of the SRAC specimens and the RAC, with the cracks in the SRAC specimens showing improvement compared to RAC. Moreover, the principal cracks on the surface of R-100 typically exhibit a steeper angle in relation to the vertical axis compared to those in N-100.

In concrete, the ITZ between the hardened cement paste with aggregates contributes to the above behavior. Cracks invariably appear along the ITZ, which is typically the weakest part of concrete [28]. The original ITZ between the natural aggregate and the adhering old mortar and the new ITZ between the recycled aggregate and the new mortar—the latter of which is more prone to fracturing—are the two forms of ITZ found in RA, as opposed to NA [29]. SRA enhances the original ITZ with the old mortar, thus improving performance compared to RA but still lagging behind NA. Consequently, the surface of RAC and SRAC exhibits an increase in secondary cracks. Furthermore, the ITZ reduces the RAC surface’s aggregate interlocking strength, which causes the primary cracks’ inclination angle to increase.

### 3.2. Cyclic Responses

**Figure 7:**
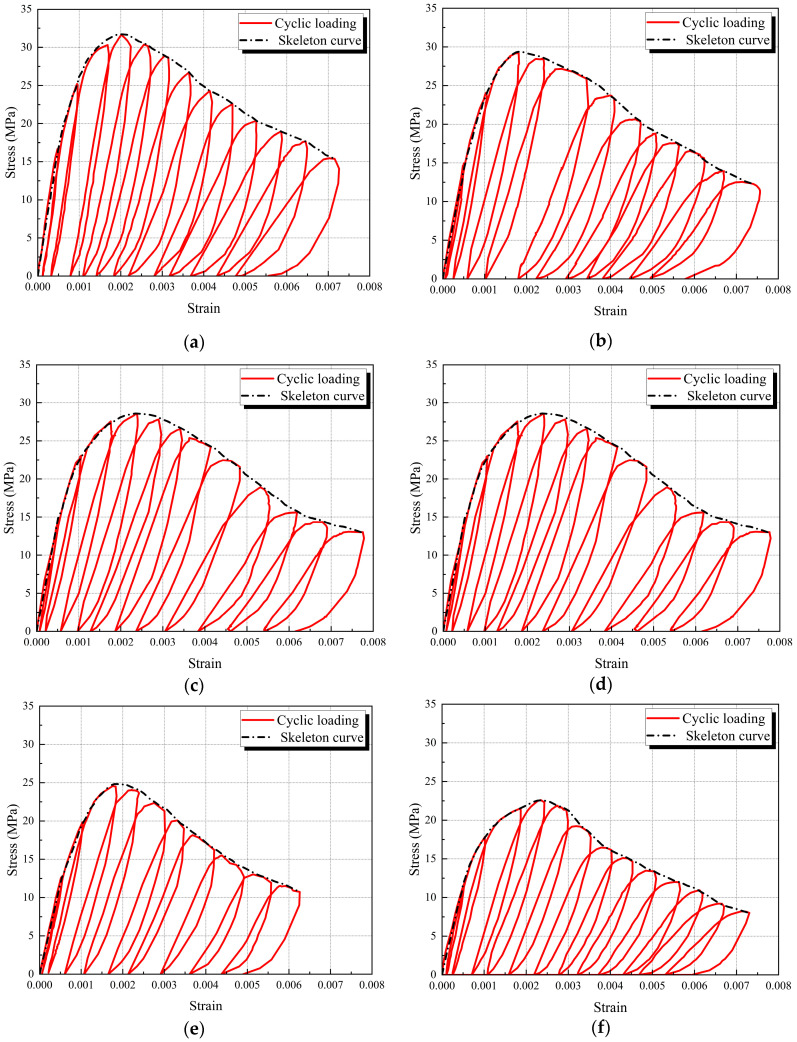
Average stress–strain curve of each group of test specimens. (**a**) N-100; (**b**) S-30; (**c**) S-50; (**d**) S-70; (**e**) S-100; (**f**) R-100.

The cyclic responses of SRAC specimens are displayed in Figure 7. By connecting the peak points of each cycle, the skeleton curve may be created using the stress–strain curves that correspond to cyclic loading. Before the stress reaches its peak, the cyclic curves almost coincide with the skeleton curve. This is because the accumulation of damage caused by cyclic loading increases with the number of cycles. Since there are fewer cycles before the peak, and the first two cycles are still in the elastic stage, the accumulation of damage is less. After the stress reaches its peak, the maximum stress of each cyclic loading decreases and eventually stabilizes. Notably, the average slope of the unloading and reloading routes falls as the maximum strain increases for each hysteresis loop under cyclic loading. This is explained by the damage that occurred as a result of the specimen’s inner microcrack growing.

**Figure 8:**
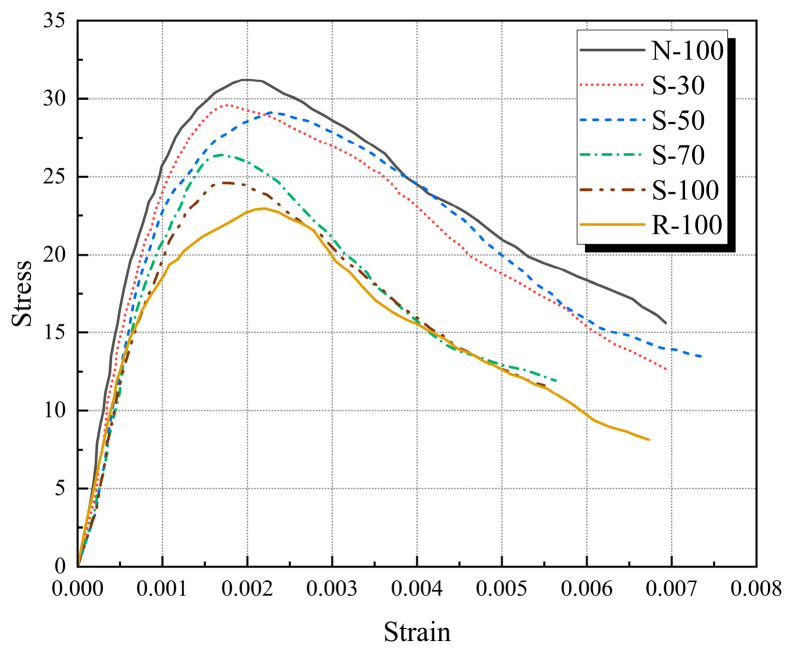
The skeleton curves of test specimens.

As can be observed in Figure 8, while comparing the test specimen skeleton curves, the ascending segment of the skeleton curve is significantly influenced by the SRA replacement ratio. The R-100 curve has the lowest slope within the elastic range, the SRAC specimen curve’s slope decreases as the SRA replacement rate increases, and the N-100 curve’s slope is noticeably higher than that of the other specimens. This trend is closely connected to the concrete’s elasticity modulus. Following the elastic range, there is more deformation after the peak under the same stress level because the slope of the SRAC specimens at peak stress and the decreasing branch diminish as the SRA replacement rate increases. Because of the high hardness and strong toughness of NA, this can reduce the speed of crack propagation and penetration in concrete, ultimately leading to a more gradual decline in the slope of the descending segment.

**Figure 9:**
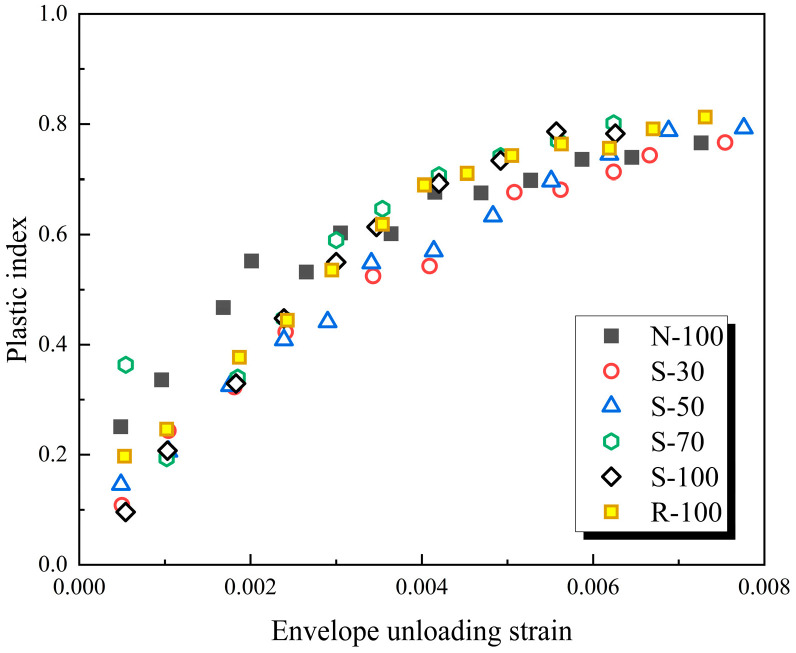
The correlation between plastic index and envelope unloading strain.

The correlation between plastic index and envelope unloading strain is shown in Figure 9. The ratio of plastic strain to envelope unloading strain, *ε*_r_/*ε*_u_, is defined as the plastic index, which ranges from 0 to 1. When the plastic index is 0, it indicates that the specimen is in an elastic state. When the plastic index is 1, the sample is in a completely plastic state. The increase in this ratio indicates an increase in the degree of damage and a greater difficulty in the concrete’s deformation recovery. Observing Figure 9, when the envelope unloading strain <0.004, the specimen’s plastic index rapidly increases with the unloading strain, and there is no significant difference between different specimens, indicating that the influence of the replacement rate of SRA is not significant during the rapid development stage of plastic damage. However, when the envelope unloading strain ≥0.004, the growth of the specimen’s plastic index begins to ease. Comparing the plastic index of the different specimens, it can be seen that the plastic indexes of R-100 and S-100 are at a higher level, while the growth of plastic index for N-100 and S-30 is relatively small. This indicates that RAC or SRAC will suffer more plastic damage than NAC, and this trend becomes increasingly apparent as the envelope unloading strain increases.

### 3.3. Axial Compressive Strength

**Figure 10:**
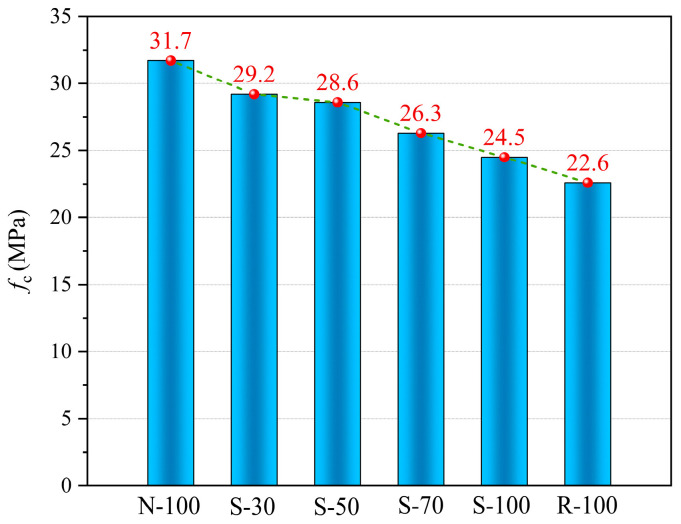
Axial compressive strength of test specimens.

The axial compressive strength of test specimens is displayed in Figure 10. It is obvious that the axial compressive strength of the specimens S-100 and R-100 dropped by 22.7% and 28.7%, respectively, in comparison to the specimen N-100. This suggests that using nano-SiO_2_ in recycled concrete can increase its axial compressive strength. The axial compressive strength of the specimens varied by 29.21%, 26.55%, 16.37%, and 8.41% compared to the specimen R-100 as the replacement rate of SRA increased. The replacement rate of SRA significantly affects the axial compressive strength of concrete. When more NA replaces SRA, it improves the inner structure of the concrete, and the inherent defects of SRA have a reduced impact on the concrete.

### 3.4. Elastic Modulus and Peak Strain

**Figure 11:**
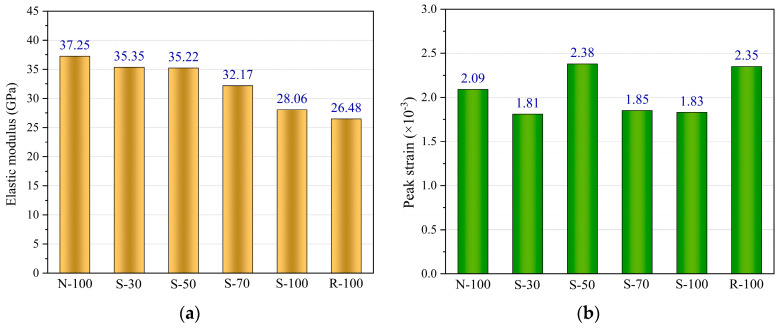
Experimental results of test specimens. (**a**) Elastic modulus of test specimens. (**b**) Peak strain of test specimens.

Figure 11a compares the elastic modulus of test specimens. It is evident that the specimens N-100 and R-100 have the highest and lowest elastic moduli, respectively. It was also found that the elastic modulus of S-100 was higher than that of R-100, but there was a significant difference compared to N-100. Moreover, the SRAC specimens’ elastic modulus decreases as the replacement rate rises. There is a slight difference in the elastic modulus between the S-30 and S-50 specimens, but overall, the elastic modulus of SRAC specimens falls as replacement rate increases. This trend coincides with the test specimens’ axial compressive strength characteristic. The ITZ of the aggregates, which will impact the concrete’s overall performance, is related to the elastic modulus of concrete. The use of NA and the application of modification procedures for RA have improved the ITZ, thereby influencing the concrete’s elastic modulus.

The peak strains of the test specimens are compared in Figure 11b. It shows that there is no apparent connection between the peak strain and the SRA replacement rate. In theory, the strain behavior of concrete is greatly influenced by the ITZ between the cement paste and the aggregate. A weak ITZ can lead to a lower elastic modulus of the concrete, which might provoke early initiation and expansion of cracks in the concrete, thereby displaying higher ultimate strain and peak strain. This may be due to the fact that under cyclic loading, the value of peak strain is also influenced by the loading system.

## 4. Discussion

### 4.1. Monotonic Stress–Strain Model

The constitutive relationships of concrete have been the subject of comprehensively experimental and theoretical investigations by numerous scholars, who have also put forth related constitutive models based on their findings. The details of current concrete constitutive models are shown in Table 5. The best constitutive model that was closest to the mechanical properties for the kind of concrete under investigation was chosen after an analysis of the available constitutive models and experimental data. The softening parameters *D* of the stress–strain curve in the Sargin [30] model are adjustable. Similarly, the parameter *C* plays a crucial role in shaping the prediction curve in the Zhou et al. [31] model. In the Guo [32] model, the ascending and descending segments of the stress–strain curve are controlled by the parameters *a* and *b*.

**Figure 12:**
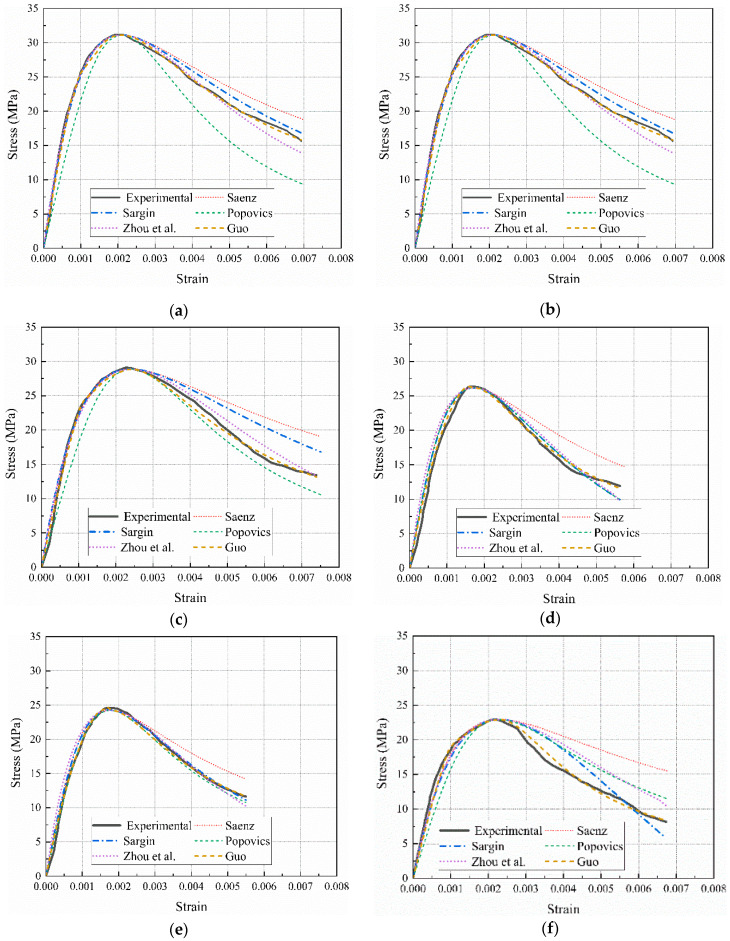
Calculation results for existing constitutive models. (**a**) N-100; (**b**) S-30; (**c**) S-50; (**d**) S-70; (**e**) S-100; (**f**) R-100 [30,31,32,33,34].

The ideal values of the model’s parameters and their correlation coefficient *R*^2^ were established by comparing the model with the results of the experiment, as indicated in Table 6. Figure 12 compares the experimental results of specimens with the current constitutive models. The results suggest that other models can accurately forecast the stress–strain curve’s ascending segment, with the exception of the Popovcis [34] model. However, the models’ predictions for the stress–strain curve’s descending segment differed significantly. The Popovcis [34] model overestimates the descending segment of the curve, whereas the Saenz [33] model shows the opposite trend. Modifying the model’s ideal parameter values, the predictions of the stress–strain curves by the Sargin [30], Zhou et al. [31], and Guo [32] models are relatively good. In contrast, the descending phase of calculation results predicted by the Guo [32] model is closest to the results from experiments, due to the presence of the controlling coefficient b for the descending phase in the model.

### 4.2. Cyclic Stress–Strain Model

#### 4.2.1. Residual Strain

**Figure 13:**
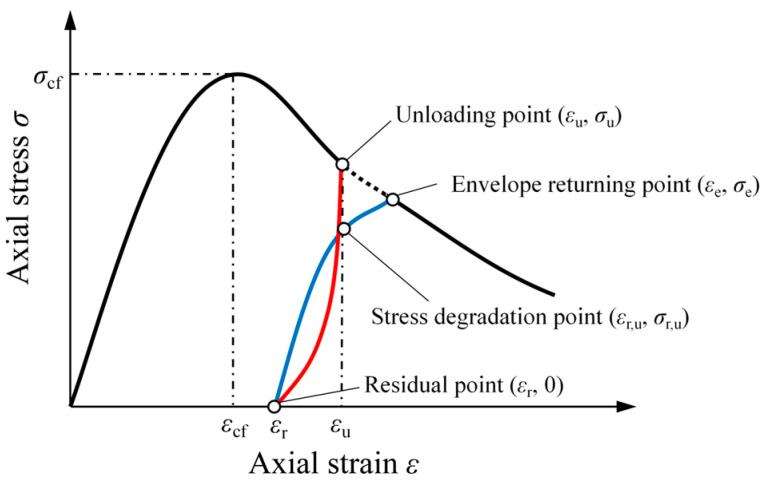
Typical hysteresis loop for SRAC specimens under cyclic loading.

**Figure 14:**
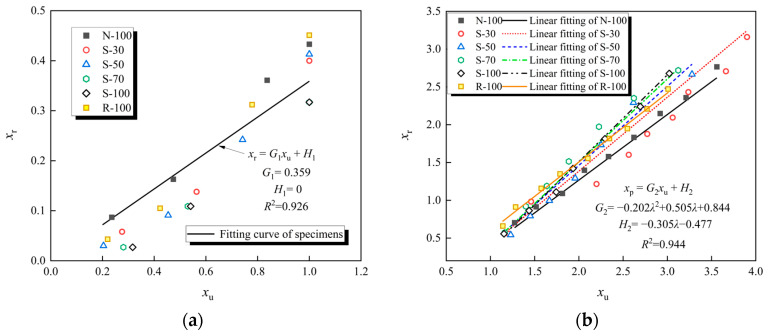
Relation between *x*_r_ and *x*_u_ of test specimens. (**a**) *x*_u_ ≤ 1; (**b**) *x*_u_ > 1.

The residual strain ε_p_ is the plastic strain after unloading from the unload point (*ε*_u_, *σ*_u_) on the envelope to the residual point (*ε*_r_, 0), as shown in Figure 13. The change in residual strain is related to the unload strain in the envelope curve. The relationship between test specimens’ residual strain (*ε*_r_) and unloading strain (*ε*_u_) is depicted in Figure 14, where *x*_r_ = *ε*_r_/*ε*_cf_, and *x*_u_ = *ε*_u_/*ε*_cf_, with *ε*_cf_ being peak strain.

The regression analysis reveals a clear linear connection between the residual strain and the unload strain. In order to explain the connection of the plastic strain and the unload strain, this study employs Li’s linear function model [35]. The relationship is separately fitted for *x*_u_ ≤ 1 and *x*_u_ > 1, taking into account the impact of SRA replacement rate. The relation between *x*_r_ and *x*_u_ of test specimens can be seen in Figure 14a,b. By fitting the data in Table 7, the formula for the SRA replacement rate (*λ*) in terms of *G*_1_, *G*_2_, *H*_1_, and *H*_2_ has been derived. The equation expressions are as follows:

(1) $$x_{r}=\left\{\begin{array}{c} G_{1}x_{u}+H_{1}\;x_{u}\leq 1\\ \;G_{2}x_{u}+H_{2}\;x_{u}>1\; \end{array}\right.$$

in which *G*_1_, *G*_2_, *H*_1_, and *H*_2_ are fitted from the data of this experiment.

*G*_1_ = 0.359, *H*_1_ = 0 (*R*^2^ = 0.914)
*G*_2_ = −0.202*λ*^2^ + 0.505*λ* + 0.844, *H*_2_ = −0.305*λ*−0.477 (*R*^2^ = 0.944)

#### 4.2.2. Stress Degradation

**Figure 15:**
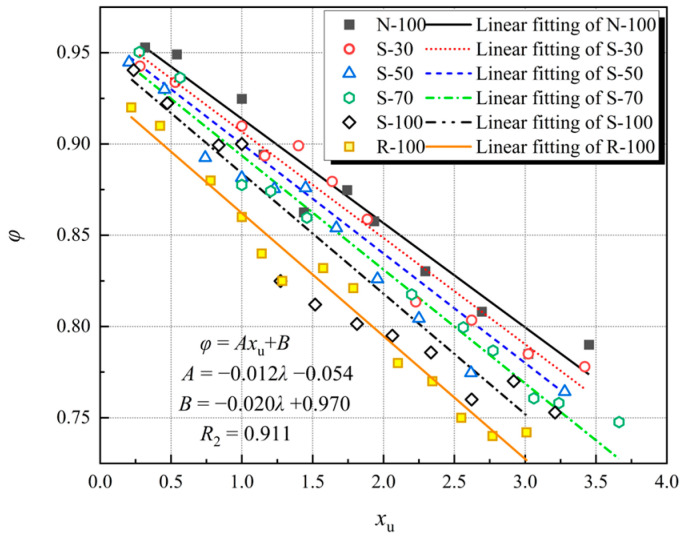
Relation curves between stress degradation rate (*φ*) and *x*_u_.

As shown in Figure 13, the stress cannot exceed the unloading stress when the reloading curve reaches the strain of the preceding unloading level. The specimen clearly exhibits stress deterioration due to the loading and unloading of cyclic stress, depicted in Figure 15. As the ratio of the stress value at the strain of the unloading point during each cycle process to the stress at the unloading point, the degradation rate may be used to express the degree of stress degradation. The equation [36] is:

(2) $$\varphi =\frac{\sigma_{u}}{\sigma_{r,u}}$$

in which *φ* represents the stress degradation percentage; *σ*_u_ represents the stress at the unloading point; *σ*_r,u_ represents the stress at the stress degradation point.

The trend of stress degradation rate is similar for different specimens. In the first three cycles before the stress reaches its peak, test specimens still retain some elasticity, making it easier for the stress to recover to the unloading point stress during the reloading phase, resulting in less stress degradation. As the quantity of cycles increases, the stress continues to degrade. Comparing the curves in Figure 14, it is found that as the SRA replacement rate increases, the stress degradation rate gradually decreases. Stress degradation increases in severity with decreasing stress degradation rate. This indicates that as the SRA replacement rate increases, it becomes more difficult for concrete to maintain its strength after being stressed.

Simplifying the analysis of the stress degradation curve of SRAC specimens, the function is approximately represented by the equation shown in Equation (3). The equation fitted is as follows:

*φ* = *Ax*_u_ + *B* (3)

in which *A* and *B* are fitted from the data of this experiment. Table 8 displays the fitted parameters *A* and *B* of the test specimens.

*A* = −0.012*λ* − 0.054, *B* = −0.02*λ* + 0.97 (*R*^2^ = 0.911)

#### 4.2.3. Envelope Returning Point

**Figure 16:**
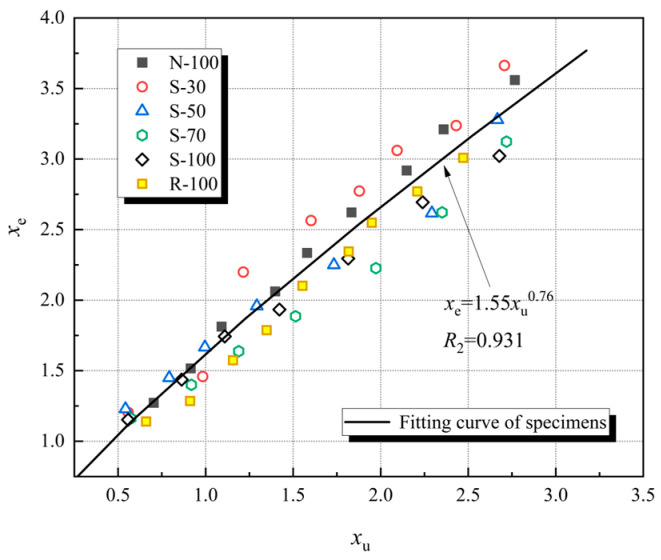
Relation between *x*_e_ and *x*_u_ of test specimens.

Figure 16 illustrates the relation between envelope returning point (*x*_e_) and unloading point (*x*_u_) of test specimens, where *x*_e_ = *ε*_e_/*ε*_cf_. With the increase in unloading strain, the envelope returning point exhibits a trend similar to the residual point. The relationship between *x*_e_ and *x*_u_ follows a power function.

(4) $$x_{e}=mx_{u}^{n}$$

in which *m* and *n* are parameters determined by experimental data. Table 9 presents the fitting parameters.

*m* = 1.55, *n* = 0.76 (*R*^2^ = 0.931)

#### 4.2.4. Unloading Path

In the cyclic stress–strain model, the unloading path refers to the behavior of concrete as it is relieved from stress during a loading cycle, demonstrating how concrete returns to its original state. This path is crucial for understanding the elastic and plastic deformation characteristics of concrete under cyclic stress. The parameters *A*_u_ and *B*_u_ of the unloading curve obtained from the experiment are used to assume that the unloading path obeys the mathematical formula given in Equation (5).

(5) $$y=y_{u}A_{u}{\left(\frac{x-x_{r}}{x_{u}-x_{r}}\right)}^{B_{u}}$$

in which *A*_u_ and *B*_u_ are calculated from the experimental data. Fitted parameters *a*_u_, *b*_u_, and *c*_u_ are shown in Table 10.

$$A_{u}=1.0,\;B_{u}=a_{u}x_{r}+b_{u}\sqrt{x_{r}}+c_{u}\;a_{u}=-0.165\lambda^{2}-0.115\lambda -0.022,\;b_{u}=1.60,\;c_{u}=0.86\;(R^{2}=0.932)$$

#### 4.2.5. Reloading Path

There are two types of curves in the reloading path: a straight line from the residual point to the stress degradation point, and a parabola from the stress degradation point to the envelope reloading point. The first branch is considered to be linear, and the second is taken to follow a power function:

(6) $$y=\frac{y_{r,u}-y_{r}}{x_{r,u}-x_{r}}(x-x_{r})+y_{r}\;,\;\;x_{r}<\;x<x_{r,u}$$

(7) $$y=y_{r,u}A_{r}{\left(\frac{x-x_{r,u}}{x_{e}-x_{r,u}}\right)}^{B_{r}}\;,\;x_{r,u}<x\leq x_{e}$$

in which *A*_r_ and *B*_r_ are determined using the experimental data. Fitted parameters *a*_r_, *b*_r_, and *c*_r_ are shown in Table 11.

$$A_{r}=1.0,\;B_{r}=a_{r}x_{e}+b_{r}\sqrt{x_{e}}+c_{r}\;a_{r}=-0.050\lambda^{2}-0.291\lambda -0.012,\;b_{r}=0.07\lambda^{2}-0.02\lambda +0.14,\;c_{r}=0.90\;(R^{2}=0.945)$$

#### 4.2.6. Verification

**Figure 17:**
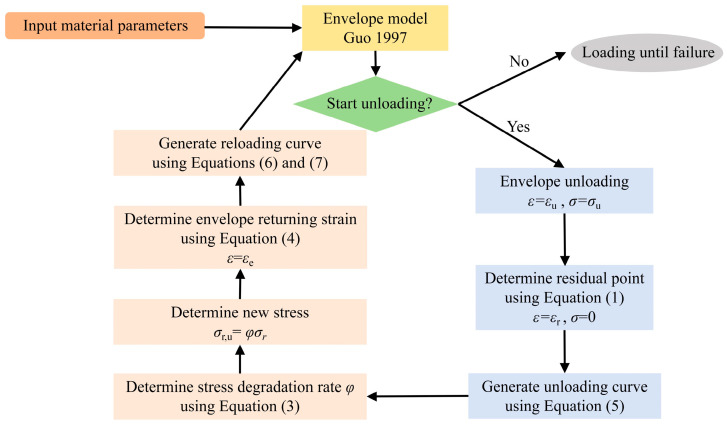
Diagram for creating stress–strain curves under cyclic loading [27].

Figure 17 shows the process of creating stress–strain curves under cyclic loading. Equations (1)–(7) form a complete constitutive model for different replacement rates of SRA under cyclic compressive load, where setting the SRA replacement rate to zero allows the model to also be applicable to normal concrete. The envelope model adopts the Guo [27] model and inputs material parameters. The envelope unloading point (*ε*_u_, *σ*_u_) must be determined before stress–strain curves under cyclic loading may be obtained. According to the envelope unloading point, Equation (1) can be used to determine the residual point (*ε*_r_, 0). The unloading path between the two points can be obtained by Equation (5) to generate the unloading curve. Subsequently, the proposed model uses Equation (3) to determine stress degradation rate φ and the stress degradation point (*ε*_r,u_, *σ*_u_). Afterward, Equation (4) is applied to determine the envelope returning point. The reloading curve can be generated from the unloading point to the returning point by using Equations (6) and (7). Consequently, it obtains the loading cycle hysteresis curve.

**Figure 18:**
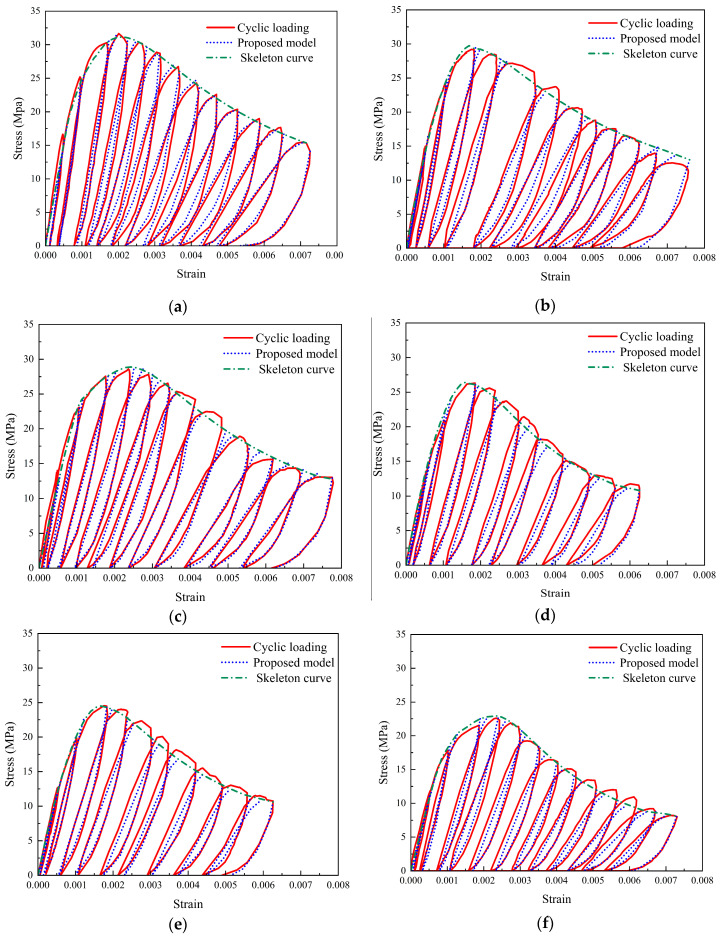
Comparison between the calculated and experimental results. (**a**) N-100; (**b**) S-30; (**c**) S-50; (**d**) S-70; (**e**) S-100; (**f**) R-100.

As illustrated in Figure 18, the cyclic curves of SRAC specimens were calculated using the model proposed in this study and compared with the results of the experiments. Generally, the predicted curves are correlated to the experimental data, indicating that the proposed model can accurately reflect the cyclic behavior of SRAC specimens.

**Figure 19:**
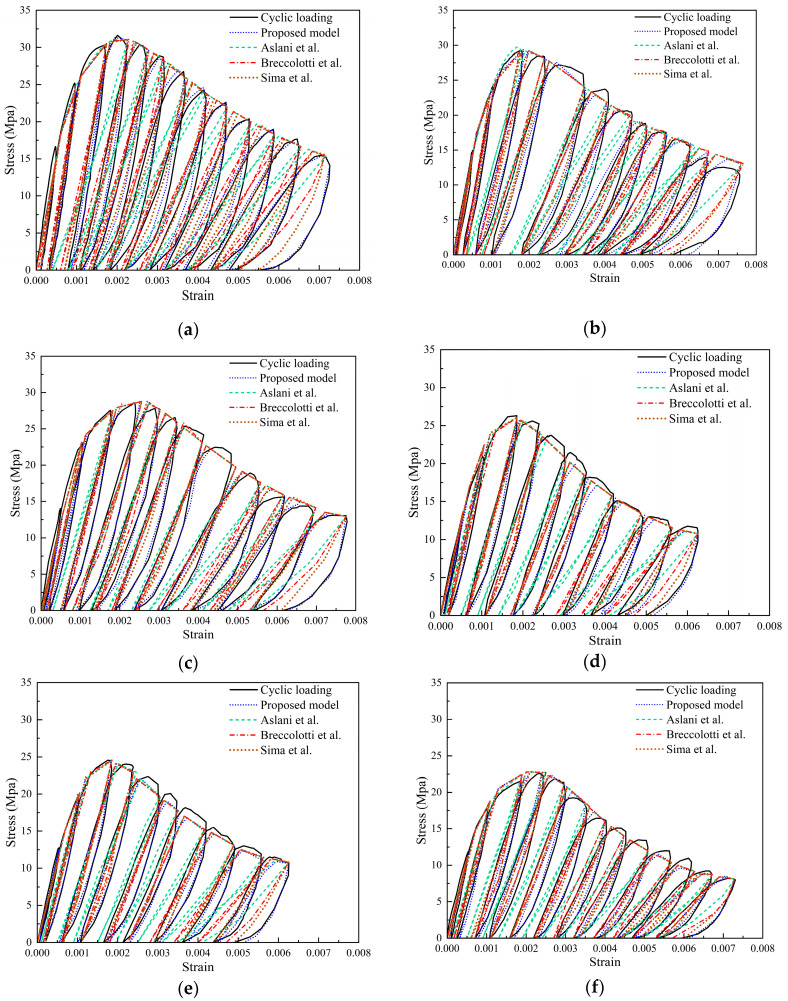
The comparison between the proposed model and existing models. (**a**) N-100; (**b**) S-30; (**c**) S-50; (**d**) S-70; (**e**) S-100; (**f**) R-100 [37,38,39].

The comparison between the proposed model and existing models can be seen in Figure 19. The existing models for comparison are as follows: the Aslani model [37], which focuses on the stress–strain relationship of concrete under cyclic loading. This model accounts for the cumulative effect of damage and the influence of initial cracks on the material response. The Breccolotti model [38] integrates the nonlinear compressive behavior of concrete, crack development, and closure effects, and attempts to describe the energy dissipation mechanism of concrete during loading-unloading cycles. Compared to the above models, the Sima model [39] adopts more comprehensive approach, specifically focusing on describing the failure mechanisms of concrete and the impact of crack development on its mechanical properties. Observing Figure 19 reveals significant differences in the computational results between the Aslani model [37] and Breccolotti model [38], especially in R-100 and S-100. Since the Sima model [39] takes into account crack development, residual strength, and stiffness changes, its computational results are closer to experimental outcomes. The proposed model in this paper, being fitted through experimental results, is the most precise. However, it must be acknowledged that the calculations of the other models consider more material properties and experimental data. The model presented in this paper is only applicable to the compressive cyclic behavior of SRAC and requires further experiments and model analysis in the future for enhancement and supplementation.

## 5. Conclusions

This study aims to systematically investigate the mechanical properties of SRAC specimens at various replacement rates under cyclic stress. The stress–strain curves of SRAC specimens for different SRA replacement rates were acquired by cyclic loading experiments. A constitutive model for SRAC exhibiting different replacement rates under cyclic stress is proposed based on the results of experiments.

(a) Nano-SiO_2_ modification treatment can enhance the axial compressive strength of RAC. Compared to RAC, increasing the SRA replacement rate (30%, 50%, 70%, and 100%) improved the axial compressive strength of SRAC specimens by 29.21%, 26.55%, 16.37%, and 8.41%, respectively. However, since nano-SiO_2_ cannot completely eliminate the impact of the inherent weaknesses of RA, the axial compressive strength of S-100 and R-100 is 22.7% and 28.7% lower than that of N-100.
(b) Comparing the experimental results with the existing constitutive models, the Guo model’s calculated results are the closest to the experimental results due to the presence of control coefficients *a* and *b*.
(c) The proposed stress–strain model of SRAC specimens regarding the SRA replacement rates under cyclic stress has relatively high computational accuracy and can be utilized to accurately forecast SRAC’s cyclic behavior at the structural and component levels.

## Figures and Tables

**Table 1:** Basic properties of P.O 42.5 Ordinary Portland Cement.

Compressive Strength (MPa)		Flexural Strength (MPa)		Specific Surface Area	Bulk Density	Density
3 Day	28 Day	3 Day	28 Day	(cm^2^/g)	(g/cm^3^)	(g/cm^3^)
16.0	42.5	3.5	6.5	360	1.3	3.11

**Table 2:** Physical properties of nano-SiO_2_ solution.

Particle Size	Color	Nano-SiO_2_ Content	pH	Density	NaO_2_ Content
(nm)		(%)		(g/cm^3^)	(%)
12	White	>99.9	10.3	1.2	0.38

**Table 3:** The performance of aggregates.

Types	Grade (mm)	Water Absorption (%)	Crushing Index (%)
NA	5–20	2.15	9.35
RA	5–20	6.06	15.39
SRA	5–20	4.77	13.04

**Table 4:** The mix proportion of test specimens (kg/m^3^).

Specimen	Replacement Rate (%)	Sand	Water	Compensation Water	Cement	NA	SRA	RA
N-100	0	688	180	8.26	500	1032	0	0
S-30	30	688	180	20.83	500	722	310	0
S-50	50	688	180	29.21	500	516	516	0
S-70	70	688	180	37.59	500	310	722	0
S-100	100	688	180	50.16	500	0	1032	0
R-100	100	688	180	74.86	500	0	0	1032

**Table 5:** Current constitutive models.

Model	Calculation Formula
Saenz [33]	$\sigma =\frac{E_{0}\varepsilon }{1+\left(\frac{E_{0}}{E_{S}}-2\right)\frac{\varepsilon }{\varepsilon_{0}}+{\left(\frac{\varepsilon }{\varepsilon_{0}}\right)}^{2}}$
Sargin [30]	$\sigma =\sigma_{0}\frac{\frac{E_{0}}{E_{S}}\frac{\varepsilon }{\varepsilon_{0}}+(D-1){\left(\frac{\varepsilon }{\varepsilon_{0}}\right)}^{2}}{1+\left(\frac{E_{0}}{E_{S}}-2\right)\frac{\varepsilon }{\varepsilon_{0}}+D{\left(\frac{\varepsilon }{\varepsilon_{0}}\right)}^{2}}$
Popovics [34]	$\sigma =\sigma_{0}\frac{\varepsilon }{\varepsilon_{0}}\frac{n}{n-1+{\left(\frac{\varepsilon }{\varepsilon_{0}}\right)}^{n}}$ *n* = 0.4 × 10^−3^*σ*_0_ + 1.0, and *σ*_0_ is in psi (1 MPa = 145 psi)
Zhou et al. [31]	$\sigma =\frac{4\sigma_{0}}{{\left(1+C\right)}^{2}}\left[e^{-\frac{\varepsilon }{\varepsilon_{0}}\ln\left(\frac{2}{1-C}\right)}+C\right]\left[1-e^{-\frac{\varepsilon }{\varepsilon_{0}}\ln\left(\frac{2}{1-C}\right)}\right]$
Guo [32]	$\begin{array}{l} \mathrm{For}\;0\leq \frac{\varepsilon }{\varepsilon_{0}}<1,\;\frac{\sigma }{\sigma_{0}}=a\frac{\varepsilon }{\varepsilon_{0}}+(3-2a){\left(\frac{\varepsilon }{\varepsilon_{0}}\right)}^{3}\\ \mathrm{For}\;\frac{\varepsilon }{\varepsilon_{0}}\geq 1,\;\frac{\sigma }{\sigma_{0}}=\frac{\frac{\varepsilon }{\varepsilon_{0}}}{b{\left(\frac{\varepsilon }{\varepsilon_{0}}-1\right)}^{2}+\frac{\varepsilon }{\varepsilon_{0}}} \end{array}$

Note: *σ* and *σ*_0_ are the concrete’s stress and peak stress, respectively; *ε* and *ε*_0_ are the concrete’s strain and peak strain, respectively; *E*_0_ is concrete’s initial elastic modulus; *E*_s_ = *σ*_0_/*ε*_0_ is the secant modulus between the origin point and peak point (*σ*_0_, *ε*_0_).

**Table 6:** Fitting parameters of the constitutive model.

Model		N-100	S-30	S-50	S-70	S-100	R-100
Sargin [30]	D	0.82	0.93	0.75	0.65	0.74	0.24
	*R* ^2^	0.988	0.970	0.984	0.943	0.978	0.916
Zhou et al. [31]	C	0.05	0.09	0.03	0.01	0.01	0.01
	*R* ^2^	0.988	0.916	0.969	0.871	0.916	0.836
Guo [32]	a	2.08	1.98	2.08	1.97	1.96	2.18
	*R* ^2^	0.988	0.989	0.966	0.975	0.989	0.968
	b	0.60	0.49	0.85	0.8	0.75	1.38
	*R* ^2^	0.998	0.958	0.974	0.992	0.993	0.983

**Table 7:** Fitted parameters *G*_1_, *G*_2_, *H*_1_, and *H*_2_ of test specimens.

Specimen	λ	*G*_1_	*H*_1_	*R* ^2^	*G*_2_	*H*_2_	*R* ^2^
N-100	0	0.597	−0.089	0.980	0.867	−0.468	0.996
S-30	0.3	0.404	−0.086	0.999	0.901	−0.562	0.968
S-50	0.5	0.486	−0.098	0.950	1.097	−0.691	0.980
S-70	0.7	0.408	−0.096	0.998	1.113	−0.631	0.987
S-100	1	0.429	−0.115	0.999	1.133	−0.794	0.997
R-100	-	0.534	−0.096	0.981	0.911	−0.315	0.993

**Table 8:** Fitted parameters *A* and *B* of test specimens.

Specimen	*λ*	*A*	*B*	*R* ^2^
N-100	0	−0.054	0.969	0.952
S-30	0.3	−0.058	0.965	0.965
S-50	0.5	−0.061	0.968	0.965
S-70	0.7	−0.061	0.956	0.976
S-100	1	−0.067	0.942	0.913
R-100	-	−0.074	0.977	0.929

**Table 9:** Fitted parameters *m* and *n* of test specimens.

Specimen	*λ*	*m*	*n*	*R* ^2^
N-100	0	1.65	0.76	0.994
S-30	0.3	1.61	0.78	0.971
S-50	0.5	1.52	0.82	0.997
S-70	0.7	1.43	0.81	0.960
S-100	1	1.53	0.80	0.978
R-100	-	1.45	0.82	0.990

**Table 10:** Fitted parameters *a*_u_, *b*_u_, and *c*_u_ of test specimens.

Specimen	*λ*	*a*_u_	*b*_u_	*c*_u_	*R* ^2^
N-100	0	−0.03	1.59	0.91	0.945
S-30	0.3	−0.06	1.59	0.88	0.959
S-50	0.5	−0.14	1.61	0.89	0.978
S-70	0.7	−0.22	1.63	0.85	0.982
S-100	1	−0.29	1.65	0.82	0.989
R-100	-	−0.40	1.68	0.79	0.965

**Table 11:** Fitted parameters *a*_r_, *b*_r_, and *c*_r_ of test specimens.

Specimen	*λ*	*a*_r_	*b*_r_	*c*_r_	*R* ^2^
N-100	0	−0.13	0.14	0.82	0.924
S-30	0.3	−0.19	0.13	0.85	0.947
S-50	0.5	−0.27	0.16	0.91	0.969
S-70	0.7	−0.38	0.15	0.98	0.985
S-100	1	−0.45	0.19	1.03	0.979
R-100	-	−0.51	0.20	1.06	0.983

## Data Availability

The data presented in this study are available from the corresponding author upon request.
